# Pattern of Use of Biosimilar and Originator Somatropin in Italy: A Population-Based Multiple Databases Study During the Years 2009–2014

**DOI:** 10.3389/fendo.2018.00095

**Published:** 2018-03-13

**Authors:** Ilaria Marcianò, Ylenia Ingrasciotta, Francesco Giorgianni, Valentina Ientile, Alessandro Chinellato, Daniele Ugo Tari, Rosa Gini, Salvatore Cannavò, Maurizio Pastorello, Salvatore Scondotto, Pasquale Cananzi, Giuseppe Traversa, Francesco Trotta, Valeria Belleudi, Antonio Addis, Gianluca Trifirò

**Affiliations:** ^1^Unit of Clinical Pharmacology, A.O.U. Policlinico “G. Martino”, Messina, Italy; ^2^Department of Biomedical and Dental Sciences and Morphofunctional Imaging, University of Messina, Messina, Italy; ^3^Pharmaceutical Service, Local Health Authority (ULSS9), Treviso, Italy; ^4^Caserta-1 Local Health Service, Caserta, Italy; ^5^Agenzia regionale di sanità della Toscana, Florence, Italy; ^6^Endocrinology, Department of Adult and Childhood Human Pathology G. Barresi, University of Messina, Messina, Italy; ^7^Department of Pharmacy, Palermo Local Health Unit, Palermo, Italy; ^8^Department of Epidemiologic Observatory, Health Department of Sicily, Palermo, Italy; ^9^Sicilian Regional Centre of Pharmacovigilance, Servizio 7-Farmaceutica, Health Department of Sicily, Palermo, Italy; ^10^Pharmacoepidemiology Unit, National Centre for Epidemiology, Italian National Institute of Health, Rome, Italy; ^11^Department of Epidemiology, Lazio Regional Health Service, Rome, Italy; ^12^Department of Medical Informatics, Erasmus Medical Centre, Rotterdam, Netherlands

**Keywords:** somatropin, health-care administrative databases, biosimilar, drug-utilization study, pattern of use

## Abstract

**Purpose:**

Somatropin [recombinant growth hormone (rGH)] is approved in children and adults for several conditions involving growth disturbances and the corresponding biosimilar is available in Italy since 2006. No population-based data are available on the pattern of rGH use in Italian clinical practice. This study aimed at exploring the pattern of biosimilar and originator rGH use in six Italian centers, where different policy interventions promoted biosimilar use.

**Methods:**

This population-based, drug-utilization study was conducted in the years 2009–2014, using administrative databases of Umbria, Tuscany, and Lazio Regions and Local Health Units of Caserta, Treviso, and Palermo. Naïve rGH users were characterized, and prevalence of use and discontinuation were assessed over time.

**Results:**

Among 6,785 patients treated with rGH during the study years, 4,493 (66.2%) were naïve users (males/females = 1.3), mostly affected by GH deficiency. The prevalence of rGH use increased from 2009 to 2010, remaining stable thereafter, but it was heterogeneous across centers (twofold higher prevalence of use in center n.2 than centers n.4 and 1 in 2014). Biosimilar rGH uptake increased over time but was low (7.8% in 2014) and heterogeneous as well. Discontinuation of rGH therapy occurred in 54.0% of naïve users, more frequently in females than males (58.1 vs. 50.9%). During the first year of treatment, discontinuation was frequent (39.9%), but no statistically significant differences were observed in treatment persistence for biosimilar vs. originator rGH (*p* > 0.05).

**Conclusion:**

Geographical heterogeneity in the prevalence of rGH use was observed. Similarly, the biosimilar rGH uptake was low and variable across centers. Post-marketing monitoring is required to continuously monitor the benefit-risk profile of rGH, thus guaranteeing greater savings than only promoting lowest cost rGH.

## Key Points

- A remarkable geographical heterogeneity in the prevalence of somatropin use was observed.
- The uptake of biosimilar somatropin was low and heterogeneous across centers, probably due to the different regional health-care policies.
- Discontinuation of somatropin therapy was frequent, but no statistically significant differences were observed for biosimilar vs. originator rGH.

## Introduction

Somatropin is a biological product containing recombinant growth hormone (rGH) that is approved in adults with pronounced GH deficiency as replacement therapy and for a wide range of conditions associated to growth disturbances and short stature in children, such as Turner or Prader–Willi syndrome, GH deficiency, chronic kidney disease (CKD) or in short children/adolescents born small for gestational age (SGA). On the Italian market, rGH is available in different devices, which differ for technical aspects, quantity of rGH, and costs. Since 2006, biosimilar rGH is available on the European and Italian market, while no biosimilars rGH are marketed in the United States (US).

A biosimilar is a biological drug containing a version of the active substance of an already authorized original biological drug (reference product) and its similarity to the corresponding reference product has been demonstrated through a comprehensive comparability exercise, with regards to quality characteristics, biological activity, safety, and efficacy (1).

Biosimilars can be considered as cheaper therapeutic alternatives to their reference products (2).

The national report on medicines use in Italy reported a decreasing overall consumption of rGH in 2015, but showed an increasing trend in biosimilar rGH use, compared to the previous year (+21.5% for biosimilar rGH). In addition, among the hormones-based systemic preparations, dispensed by public hospitals (excluding sex hormones), rGH ranks first for cost, with expenditure at €1.5/per capita (3) However, a relevant difference in overall rGH consumption across Italian regions has been reported (4), showing a patchy pattern throughout the country.

A “National register of rGH users” has been set up to monitor rGH prescriptions with the goal to prevent any risk associated with inappropriate use. A corresponding report is yearly published by the Italian Institute of Health, but the collection of these data is not homogeneous across the country and they are currently available only for some Regions. Results from the only published study using data from the National register estimated a prevalence rate of rGH treatment (patients <18 years) in Piedmont Region equal to around 0.9 per 1,000 inhabitants in 2004 (5), while data from the most recent yearly National report from the register showed a prevalence of rGH use around 0.2 per 1,000 inhabitants in the available Regions (6).

Recombinant growth hormone therapy requires regular, daily, subcutaneous injections. Adherence to the recommended regimens is crucial to achieve successful outcomes and to reach the final height (7), but non-adherence is a common problem in clinical practice, especially in pediatric patients (up to half of children are not fully adherent) (7–10). Furthermore, although rGH therapy is not always indicated after the final height is reached, several studies showed the negative effects of rGH discontinuation during transition age on bone and muscle mass (11–15) in case of severe GH deficiency.

To date, no population-based data about prescribing pattern of originator and biosimilar rGH in Italian routine care are available.

This population-based, multi-database study was aimed at exploring the pattern of rGH use, both biosimilar and originators, in six large Italian areas, where various health-care policy interventions were adopted in order to promote biosimilars use.

## Materials and Methods

### Data Source

This Italian, retrospective, population-based, multi-database study was conducted using data extracted from the administrative databases of Caserta, Treviso, and Palermo Local Health Units (LHUs) and Tuscany, Umbria, and Lazio Regions, covering around 25% of the whole Italian population (more than 14 million persons) during the years 2009–2014. Each center collects pharmacy claims data for dispensed drugs to the residents in the catchment areas. Thanks to unique patient identifiers (anonymized), different claims data [diagnosis at hospital discharge, reasons for health-care service payment exemptions, emergency department (ED) visits diagnosis, and other dispensed drugs reimbursed by the National Health Service (NHS)] from the six centers can be linked together. rGH prescription is associated to a therapeutic plan that is filled by specialists, who report the exact indication for use, brand name, dosing regimen, and dispensed number of packages. In Caserta and Treviso LHUs and in Lazio Region, therapeutic plans were available as electronic forms. The Anatomical Therapeutic Chemical (ATC) classification system is used to code drugs information, while the International Classification of Disease, clinical modification, ninth revision (ICD9-CM) is used to code information about the indication for use, the diagnosis at hospital discharges and the reasons for ED visits. This database network has been previously used for the postmarketing assessment of biosimilar use and has been described more in detail elsewhere (16, 17). The six centers were anonymized in all the analyses.

### Study Population

The source population included all the residents in the catchment areas of the six participating centers during the years 2009–2014 (2011–2014 in Umbria Region). From this population, all patients having at least 1 year of database history and at least one dispensing of rGH during the study years were identified.

### Study Drugs

During the years 2009–2014, the medicinal products containing rGH available on the Italian market were Genotropin^®^, Humatrope^®^, Norditropin^®^, Nutropinaq^®^, Omnitrope^®^, Saizen^®^, Somatropin Biopartners^®^, Zimoser^®^, and Zomacton^®^ (ATC: H01AC01). Omnitrope^®^ is the only biosimilar (see Table S1 in Supplementary Material). The abovementioned medicinal products are approved for several indications, ranging from inadequate endogenous growth hormone secretion in children to growth failure associated with Prader–Willi or Turner syndromes or CKD, growth disturbance in children SGA, and as replacement therapy in adults with pronounced GH deficiency. In addition, Genotropin^®^ and Omnitrope^®^ are also approved to improve growth and body composition in children with Prader–Willi syndrome, while Humatrope^®^ is the only rGH approved for the treatment of growth failure associated with Short stature HOmeoboX-containing (SHOX) gene deficiency. In 2000, orphan designation was granted by European Medicines Agency for rGH for Acquired Immune Deficiency Syndrome wasting (18).

Recombinant growth hormone is administered as daily subcutaneous injection and is available on the market as already prepared in *ad hoc* devices. In detail, the main differences across different products are related to volume of rGH (both in terms of dose range and increments), temperature for storage, requirement for reconstitution, type of injection (i.e., manual, automatic), presence/absence of needle (which could influence the patients’ compliance, especially of younger ones, and adherence to therapy), and potential immunogenicity (due to specific excipients, i.e., benzyl alcohol).

### Health-Care Policy Interventions

The Italian NHS provides Italian citizens with universal coverage for most of the health-care services and is tax-based. Regional governments are responsible for providing their population with a nationally defined package of health-care services, using a specific budget transferred by the national government (19). Within the NHS, each Region can autonomously take specific drug-related policy interventions for controlling drug expenditure. Tuscany, Lazio, and Umbria (Central Italy) are regions themselves, while Treviso, Caserta, and Palermo are respectively part of Veneto (Northern Italy), Campania, and Sicily regions (Southern Italy). In these six Regions, different health policy interventions about biosimilars were applied over time. The first Region to impose the use of biosimilars as first-line therapy in naive users was Campania, followed by Tuscany and Veneto in 2010, Umbria in 2013, and Sicily in 2014, as already previously discussed (17). In Lazio, a specific working group on biosimilars was created in 2015, but already in 2014, a specific intervention regarding rGH use recommended the use of the cheapest drug, except in case of clinically relevant reasons (therapeutic continuity, tolerability of excipients) to be documented in the therapeutic plans (20, 21). Regional drug policies concerning biosimilars in general have been described elsewhere (17), while those specifically related to rGH are listed in Table S2 in Supplementary Material.

### Data Analysis

Across the six participating centers, anonymized data on rGH users underwent quality controls through benchmarking of several parameters and were pooled.

#### Characterization of Naïve rGH Users

The Index Date (ID) was identified as the date of the first dispensed rGH during the study years. Naive rGH users (i.e., users with no dispensing of rGH within 1 year prior to the ID) were identified in each database. Naive rGH users were characterized, in terms of type of rGH dispensed at ID, patients’ demographics, comorbidities, and concomitant drugs. Homogeneity of sex and age variables among the six participating centers was tested using chi-squared and ANOVA test, respectively. The indication of use was available only in four centers (i.e., Caserta LHU, Toscana, Umbria, and Lazio Regions) for 54% of the incident users and was identified using electronic therapeutic plans, whenever available, diagnosis at hospital discharge, reasons for health-care service payment exemptions, and ED visits diagnosis any time prior to the ID. Comorbidities (in terms of both number of hospitalizations and specific diseases, e.g., diabetes mellitus, hypertension, thyroid disorders) have been evaluated within 1 year prior to ID; neoplasms-specific ICD-9CM diagnosis codes were identified within 6 months prior to the ID; concomitant drugs (antidiabetic agents, antihypertensive agents, cortisone, desmopressin, thyroid preparations, lipid modifying agents, testosterone, vitamin D and analogs) have been sought for within 3 months prior to ID. In addition, the number of ATC codes (I and V level), other than rGH, dispensed in the same period was evaluated.

#### Prevalence of rGH Use

The yearly crude and age-adjusted center-specific prevalence of rGH users per 1,000 inhabitants, together with 95% confidence intervals, was calculated during the study period, by dividing the number of patients having at least one dispensing of rGH with the number of residents in the catchment areas during the same observation period. Stratifying by center and calendar year, the percentage of biosimilar rGH users on the total of rGH users was assessed.

#### Switching Pattern

The switching pattern analyses of various rGHs within the first year after ID was performed both overall and stratifying by center, including naive rGH users with at least 1 year of observation and one dispensing of rGH during the first year after the ID. Only the first switch after the ID was considered.

#### Persistence Analysis

A time to event analysis (i.e., time to discontinuation) was performed on naïve rGH users, to assess treatment persistence over time. For each naïve rGH user, the number of days of continuous rGH treatment from the beginning of the therapy was estimated, based on the Defined Daily Dose (DDD) of rGH and the amount of dispensed rGH. Persistence to therapy was assessed based on the maximum allowed treatment gap, defined as the time between the last day covered by rGH treatment and the time to the next refill. Naïve rGH users were considered discontinuers if they had at least one treatment gap exceeding 60 days. For discontinuers, the time to discontinuation was calculated as the number of days between ID and the last day covered by rGH treatment. Kaplan Meier analysis was carried out and results were stratified by sex, by sex, and age classes (≤11; 12–17; 18–25; and >25 years old), and by medicinal product. Follow-up of naïve rGH users was censored if patients were still on therapy at the end of the study, in case of death or no availability of further data, whichever came first. Using dispensing database, diagnosis at hospital discharge and reasons for health-care service payment exemptions, we investigated potential reasons for discontinuation of rGH therapy. Discontinuers who restarted rGH treatment after the initial interruption (i.e., intermittent users) were first identified. Thereafter, excluding intermittent users, we identified discontinuers having a diagnosis of cancer or diabetes from 3 months prior to discontinuation to the end of follow-up or being hospitalized after discontinuation. Among the remaining discontinuers, we identified early discontinuers (i.e., those who discontinued within 6 months from the ID), stratifying by age classes.

#### Subgroup and Sensitivity Analyses

In order to validate the main persistence analysis conducted on all naïve rGH users, a subgroup analysis was performed on naïve rGH users affected by GH deficiency, using a maximum allowed treatment gap of 60 days. Naive users with other indications of use were excluded due to their low number.

Sensitivity analyses were conducted: (i) within the first year of treatment after ID (maximum allowed treatment gap of 60 days), stratifying by age classes; (ii) by defining naïve rGH users as discontinuers if they had a treatment gap exceeding 2 years after the discontinuation date.

#### Ethics Statement

The study was conducted in the context of the project “Assessment of Short and Long Term Risk–Benefit Profile of Biologics Through Healthcare Database Network in Italy” (RF-2010-2320172), which was funded by the Italian Ministry of Health. The study protocol was approved by the Ethical Committee of the Academic Hospital of Messina, which was the coordinator center (minutes n.9/2014, 21st July 2014).

## Results

**Figure 1:**
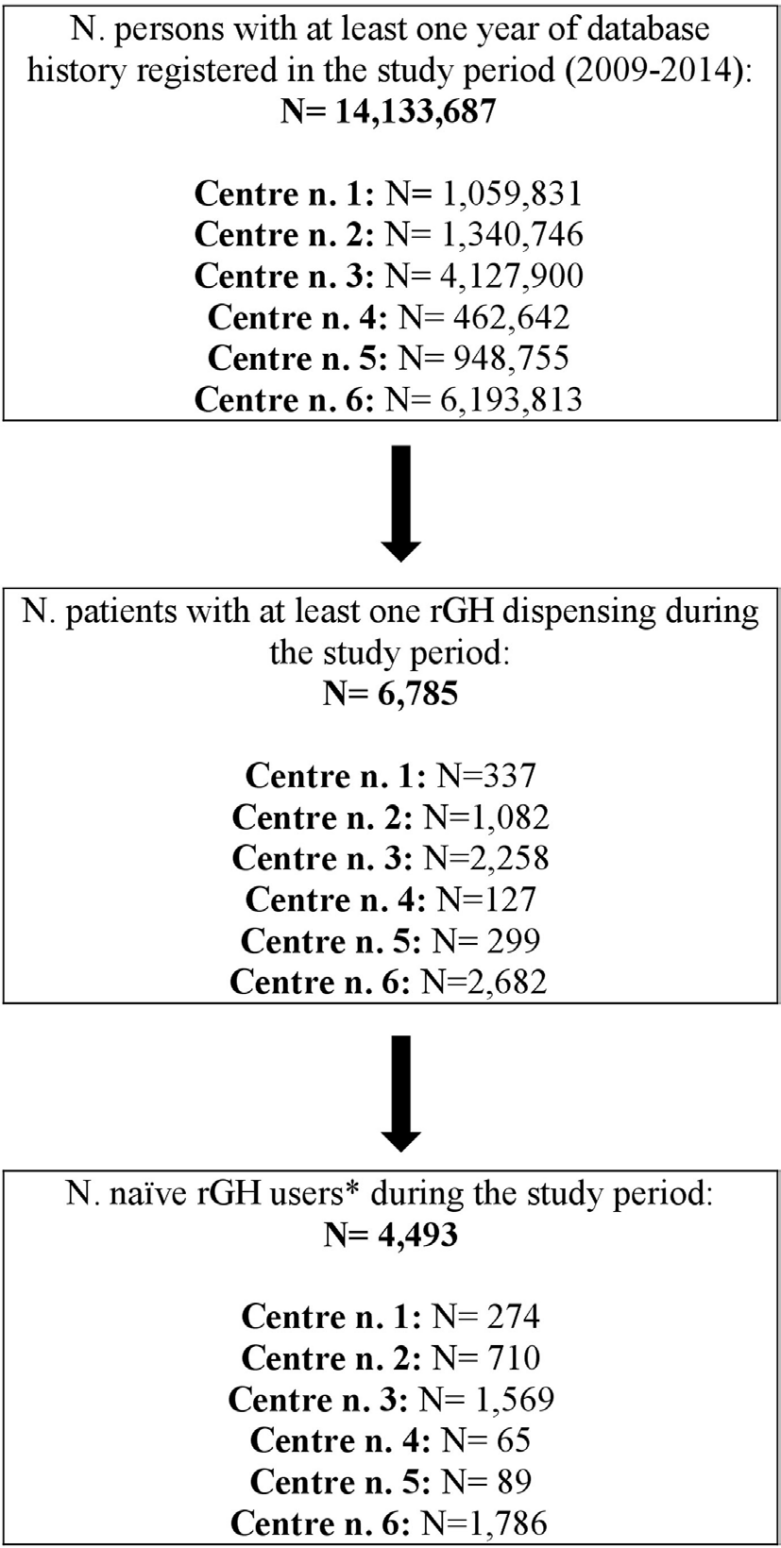
Identification of rGH users in the six participating centers. Legend: rGH, recombinant growth hormone (i.e., somatropin). *Naïve rGH users: rGH users without any rGH dispensing within 1 year prior to Index Date (date of first rGH dispensing during the study period).

Overall, the source population covered 14,133,687 persons with at least 1 year of database history registered in the years 2009–2014 (around 25% of the whole Italian population). Of these, 6,785 (0.05%) received at least one rGH dispensing during the study years (Figure 1). Considering rGH users, 4,493 (66.2%) were naïve. rGH treatments were more frequently received by males (males to females ratio = 1.3), in all centers but one (males/females = 0.9). Both considering males and females, most of naïve rGH users were ≤11 years old at ID (males: *N* = 1,076; 42.2%). Naïve users had a median age of 12.0 years; most of them were ≤11 years old (*N* = 2,030; 45.2%). Concerning age, a slightly statistically significant difference was observed among the six participating centers (*p* < 0.05); therefore, the following analyses were conducted taking into account age categories as stratification factor.

The indication of use was available for 2,430 naïve rGH users (54.1%) and the most frequent was the GH deficiency (*N* = 2,201; 88.8%). As first prescription, rGH users were more likely to receive reference product than biosimilar, especially in centers n. 6 and 2 (94 vs. 6%) (Table 1; Figure S1 in Supplementary Material). Almost half of naïve rGH users had been hospitalized at least once within 1 year prior to the ID (*N* = 2,104; 46.9%), more frequently with diagnosis related to the primary disease (e.g., pituitary gland disorders, symptoms concerning development). Among considered comorbidities, 13.9% of naïve rGH users were affected by hypertension, followed by 13.3 and 7.3% suffering from thyroid disorders and diabetes, respectively. Within 6 months prior to the ID, 0.8% of naïve rGH users had at least one diagnosis of benign neoplasm, while 1.3% of naïve rGH users had at least one registered diagnosis of malignant neoplasm.

**Table 1:** Characterization of naïve rGH users^a^ at baseline, stratified by center.

	Center n. 1 *N* = 274 (%)	Center n. 2 *N* = 710 (%)	Center n. 3 *N* = 1,569 (%)	Center n. 4 *N* = 65 (%)	Center n. 5 *N* = 89 (%)	Center n. 6 *N* = 1,786 (%)	Total *N* = 4,493 (%)
Sex							
Male	148 (54.0)	437 (61.6)	871 (55.5)	31 (47.7)	55 (61.8)	1,007 (56.4)	2,549 (56.7)
Female	126 (46.0)	273 (38.4)	698 (44.5)	34 (52.3)	34 (38.2)	779 (43.6)	1,944 (43.3)
Age—median (q1–q3)	12 (9–21)	11 (8–13)	13 (10–50)	10 (4–29)	12 (9–14)	12 (9–36)	12 (9–21)

Age categories (years)							
≤11	135 (49.3)	438 (61.7)	614 (39.1)	38 (58.5)	41 (46.1)	764 (42.8)	2,030 (45.2)
12–17	62 (22.6)	225 (31.7)	411 (26.2)	7 (10.8)	29 (32.5)	447 (25.0)	1,181 (26.3)
18–25	16 (5.8)	6 (0.8)	27 (1.7)	1 (1.5)	2 (2.3)	57 (3.2)	109 (2.4)
>25	61 (22.3)	41 (5.8)	517 (33.0)	19 (29.2)	17 (19.1)	518 (29.0)	1,173 (26.1)

Indication for use—short stature due to^b^	170 (62.0)	–	802 (51.1)	–	67 (75.3)	1,391 (77.9)	2,430 (54.1)
GH deficiency	153 (90.0)	–	713 (88.9)	–	63 (94.0)	1,272 (91.5)	2,201 (88.8)
CKD	8 (4.7)	–	53 (6.6)	–	1 (1.5)	44 (3.2)	106 (4.4)
Turner syndrome	4 (2.4)	–	8 (1.0)	–	2 (3.0)	42 (3.0)	56 (2.3)
Prader–Willi syndrome	2 (1.2)	–	3 (0.4)	–	–	31 (2.2)	36 (1.5)
SGA	3 (1.7)	–	25 (3.1)	–	1 (1.5)	2 (0.1)	31 (1.3)

Index year							
2009	70 (25.5)	–	237 (15.1)	–	–	457 (25.6)	764 (17.0)
2010	35 (12.8)	115 (16.2)	301 (19.2)	10 (15.4)	–	323 (18.1)	784 (17.4)
2011	38 (13.9)	137 (19.3)	338 (21.5)	12 (18.5)	–	311 (17.4)	836 (18.6)
2012	45 (16.4)	154 (21.7)	198 (12.6)	5 (7.7)	33 (37.1)	286 (16.0)	721 (16.0)
2013	31 (11.3)	124 (17.5)	307 (19.6)	26 (40.0)	34 (38.2)	188 (10.5)	710 (15.8)
2014	55 (20.1)	180 (25.4)	188 (12.0)	12 (18.5)	22 (24.7)	221 (12.4)	678 (15.1)
Follow-up, years—median (q1–q3)^c^	3.0 (1.2–5.0)	2.3 (1.0–3.5)	3.2 (1.6–4.2)	1.9 (1.3–3.6)	1.6 (1.0–2.2)	3.6 (2.1–5.0)	3.0 (2.0–5.0)

Type of rGH							
Biosimilar	22 (8.0)	43 (6.1)	257 (16.4)	21 (32.3)	10 (11.2)	106 (6.0)	459 (10.2)
Reference product	252 (92.0)	667 (93.9)	1,312 (83.6)	44 (67.7)	79 (88.8)	1,680 (94.0)	4,034 (89.8)
N. hospitalizations							
0	141 (51.5)	698 (98.3)	737 (47.0)	49 (75.4)	56 (62.9)	708 (39.6)	2,389 (53.1)
1–2	110 (40.1)	11 (1.5)	687 (43.8)	15 (23.1)	29 (32.6)	840 (47.0)	1,692 (37.7)
>2	23 (8.4)	1 (0.1)	145 (9.2)	1 (1.5)	4 (4.5)	238 (13.3)	412 (9.2)

Comorbidities^d^							
Hypertension	29 (10.6)	22 (3.1)	305 (19.4)	4 (6.2)	11 (12.4)	255 (14.3)	626 (13.9)
Thyroid disorders	41 (15.0)	36 (5.1)	169 (10.8)	10 (15.4)	15 (16.9)	327 (18.3)	598 (13.3)
Diabetes mellitus	11 (4.0)	17 (2.4)	159 (10.1)	2 (3.1)	5 (5.6)	135 (7.6)	329 (7.3)
Neoplasms^e^	8 (2.9)	–	44 (2.7)	2 (3.1)	2 (2.2)	41 (2.3)	97 (2.2)
Malignant neoplasm	5 (1.8)	–	27 (3.8)	1 (1.5)	1 (1.1)	25 (1.4)	59 (1.3)
Benign neoplasms	3 (1.1)	–	17 (1.1)	1 (1.5)	1 (1.1)	16 (0.9)	38 (0.8)

Concomitant drugs, within 3 months prior to ID Number of distinct ATC (other than rGH)							
0	144 (52.6)	481 (67.7)	715 (45.6)	48 (73.8)	48 (53.9)	947 (53.0)	2,383 (53.0)
1	36 (13.1)	119 (16.8)	249 (15.9)	3 (4.6)	21 (23.6)	325 (18.2)	753 (16.8)
2–3	46 (16.8)	73 (10.3)	208 (13.3)	7 (10.8)	9 (10.1)	286 (16.0)	629 (14.0)
>3	48 (17.5)	37 (5.2)	397 (25.3)	7 (10.8)	11 (12.4)	228 (12.8)	728 (16.2)

ATC - I level							
A—alimentary tract and metabolism	36 (13.1)	44 (6.2)	330 (21.0)	7 (10.8)	12 (13.5)	314 (17.6)	743 (16.5)
B—blood and blood forming organs	27 (9.8)	14 (2.0)	260 (16.6)	–	3 (3.4)	116 (6.5)	420 (9.3)
C—cardiovascular system	26 (9.5)	21 (2.9)	287 (18.3)	1 (1.5)	9 (10.1)	235 (13.2)	579 (12.9)
G—genito urinary system and sex hormones	13 (4.7)	6 (0.8)	86 (5.5)	6 (9.2)	3 (3.4)	77 (4.3)	191 (4.3)
H—systemic hormonal preparations, excl. sex hormones, and insulins	53 (19.3)	58 (8.2)	240 (15.3)	13 (20.0)	17 (19.1)	245 (13.7)	626 (13.9)
J—anti-infectives for systemic use	75 (27.4)	152 (21.4)	374 (23.8)	3 (4.6)	22 (24.7)	377 (21.1)	1,033 (23.0)
L—antineoplastic and immunomodulating agents	11 (4.0)	7 (1.0)	100 (6.4)	–	2 (2.2)	27 (1.5)	147 (3.3)
M—musculo-skeletal system	20 (7.3)	9 (1.3)	152 (9.7)	2 (3.1)	4 (4.5)	125 (7.0)	312 (6.9)
N—nervous system	15 (5.5)	13 (1.8)	319 (20.3)	5 (7.7)	4 (4.5)	109 (6.1)	465 (10.3)
Others^f^	6 (2.2)	10 (1.4)	57 (3.6)	–	1 (1.1)	67 (3.8)	141 (3.1)

Drug classes							
Antihypertensive agents	24 (8.8)	19 (2.7)	259 (16.5)	1 (1.5)	9 (10.1)	209 (11.7)	521 (11.6)
Thyroid preparations	28 (10.2)	31 (4.4)	116 (7.4)	9 (13.8)	13 (14.6)	192 (10.8)	389 (8.7)
Antidiabetic agents	9 (3.3)	14 (2)	127 (8.1)	1 (1.5)	1 (1.1)	106 (5.9)	258 (5.7)
Lipid modifying agents	13 (4.7)	9 (1.3)	120 (7.6)	–	1 (1.1)	86 (4.8)	229 (5.1)
Vitamin D and analogs	12 (4.4)	12 (1.7)	73 (4.7)	1 (1.5)	9 (10.1)	57 (3.2)	164 (3.7)
Cortisone	19 (6.9)	13 (1.8)	53 (3.4)	8 (12.3)	6 (6.7)	16 (0.9)	115 (2.6)
Desmopressin	9 (3.3)	5 (0.7)	35 (2.2)	2 (3.1)	4 (4.5)	50 (2.8)	105 (2.3)
Testosterone	–	–	27 (1.7)	6 (9.2)	–	5 (0.3)	38 (0.8)

*rGH, recombinant growth hormone (somatropin); q1–q3, interquartile range; ATC, Anatomical Therapeutical Chemical Classification system; ID, Index Date; CKD, chronic kidney disease; SGA, small for gestational age*.

*^a^Naïve rGH users: rGH users without any rGH dispensing in the year prior to ID (date of first dispensing of rGH during the study period)*.

*^b^The indication of use was available only in Center n. 1, 3, 5, 6*.

*^c^Duration of follow-up is calculated from ID till the end of follow-up*.

*^d^Comorbidities were identified using hospital discharge and emergency department (ED) visits diagnoses, health-care service payment exemption reasons, and specific dispensed drugs that were registered within 1 year prior to ID*.

^e^Neoplasms were identified from hospital discharge and ED visit diagnoses (ICD9CM code: 140*-239*) within 6 months prior to ID.

*^f^Others include the following ATC I level: D (dermatologicals), P (Antiparasitic products, insecticides and repellents), S (sensory organs), and V (various)*.

Almost half of naïve rGH users had been dispensed at least one drug other than rGH within 3 months prior to the ID (*N* = 2,110; 47.0%).

**Figure 2:**
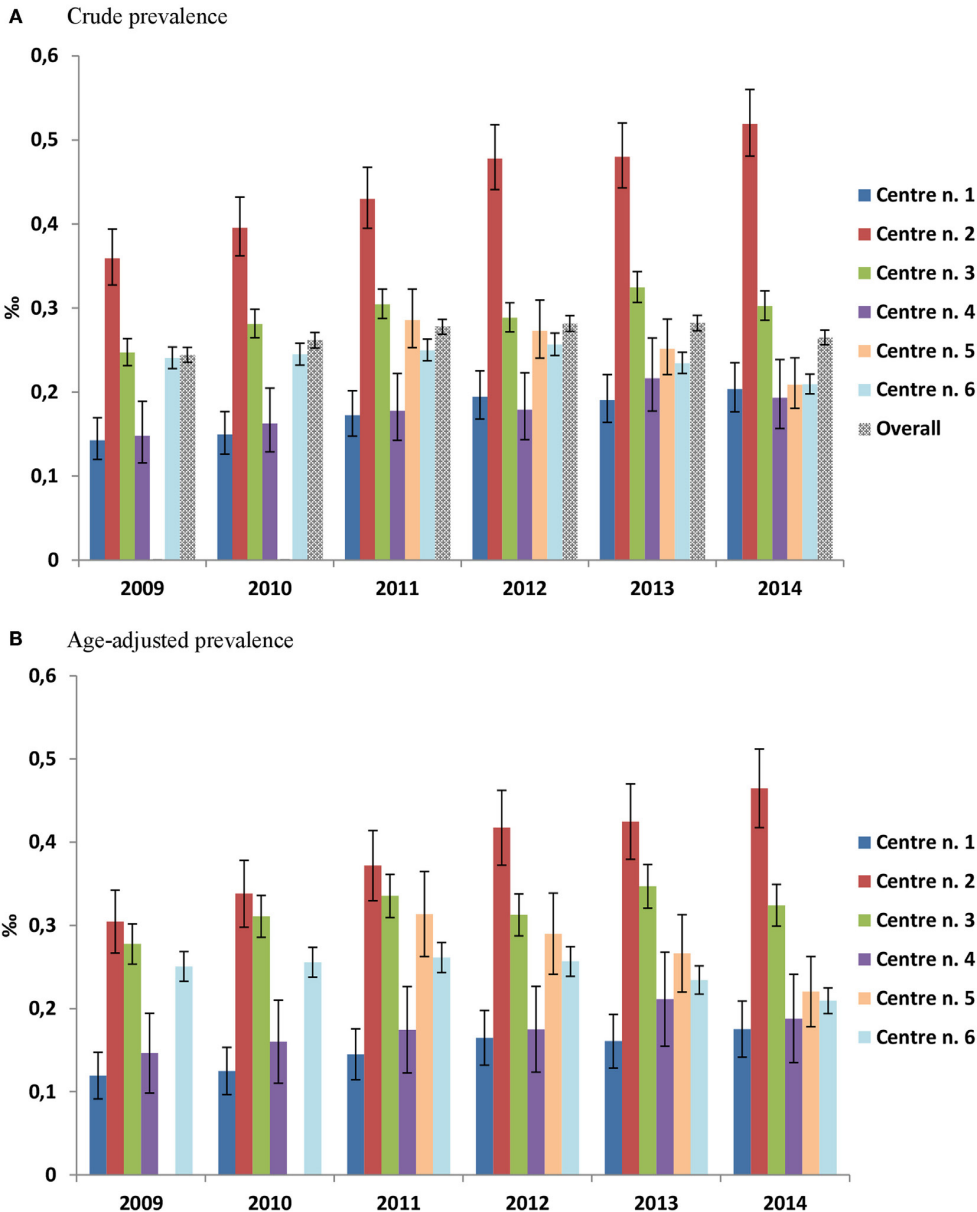
Prevalence of recombinant growth hormone users per 1,000 inhabitants, stratified by calendar year and center. **(A)** Crude prevalence of use, **(B)** age-adjusted prevalence of use.

**Figure 3:**
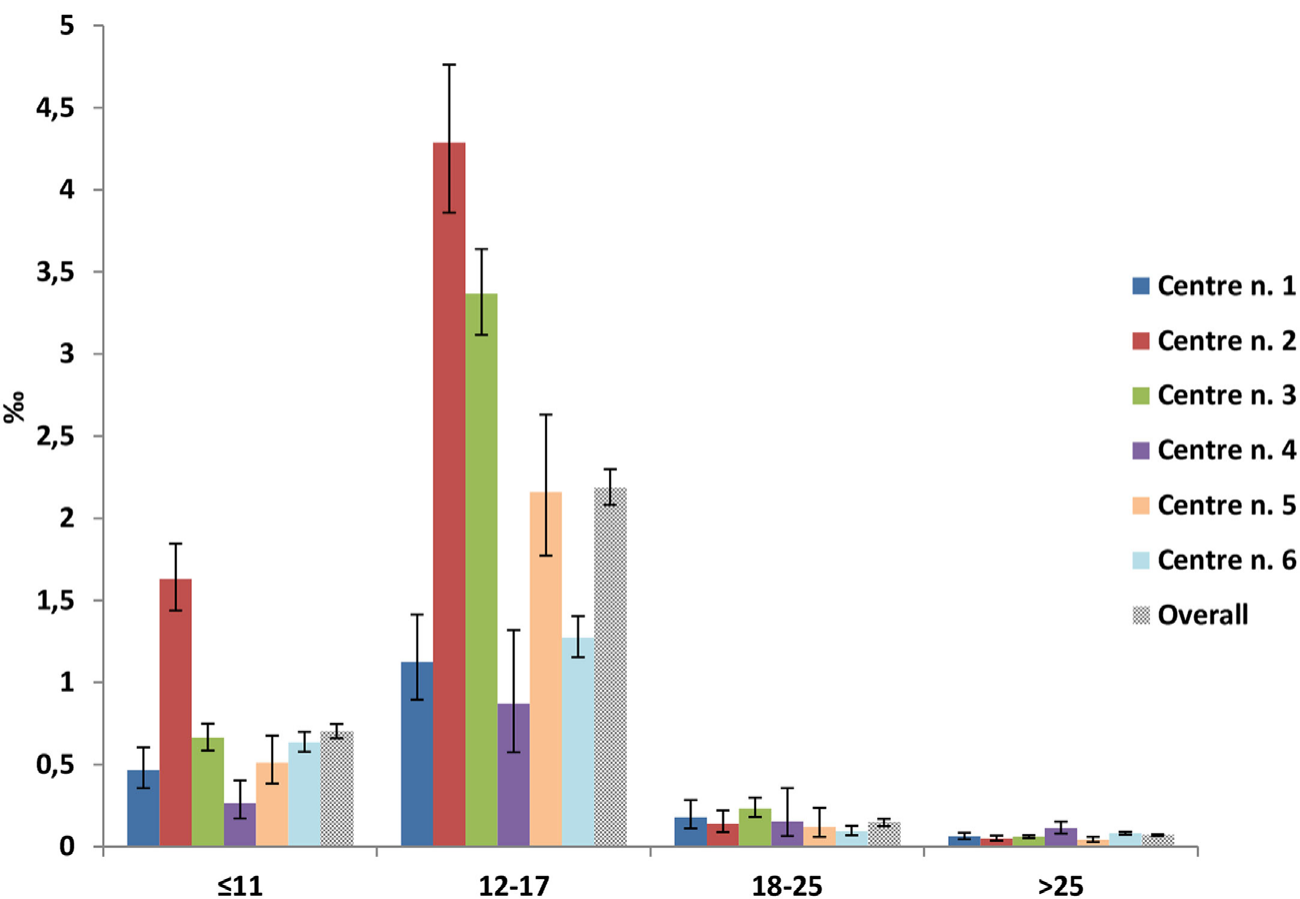
Prevalence of recombinant growth hormone users per 1,000 inhabitants in 2014, stratified by age classes and center.

Overall, the prevalence of rGH users (Figure 2) slightly increased during the first two observation years (from 0.2 per 1,000 inhabitants in 2009 to 0.3 per 1,000 inhabitants in 2010), while remaining stable thereafter. Prevalence of use was, however, heterogeneous across centers with center n. 2 reporting twofold higher prevalence of rGH use than centers n. 4 and 1 in 2014, which was up to fourfold higher in those >18 years old (Figure 3).

**Figure 4:**
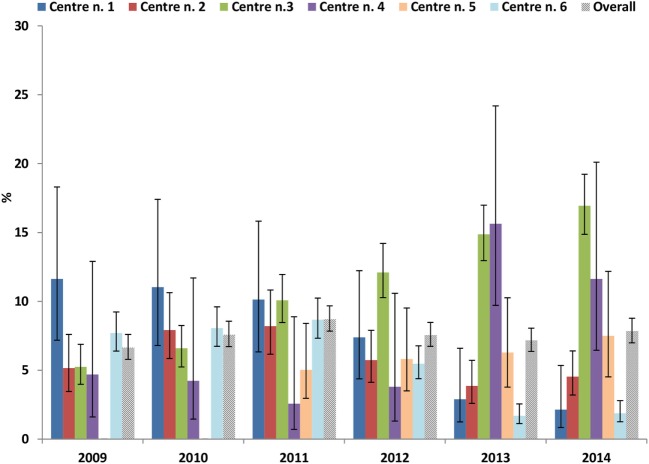
Proportions (%) of users of biosimilars recombinant growth hormone on the total of recombinant growth hormone users, stratified by calendar years and center.

Overall, the proportion of biosimilar rGH users was low and ranged from 6.6% in 2009 to 7.8% in 2014 (Figure 4). A decreasing trend was observed over time in center n. 1 (11.6 vs. 2.1%) and n. 6 (7.7 vs. 1.9%); instead, an increasing trend was observed in center n. 5 (5.0 vs. 7.5%), center n. 4 (4.7 vs. 11.6%), and even more in center n. 3 (5.2 vs. 16.9%).

In our cohort, 3,084 naïve users (68.6%) had at least 1 year of observation after the ID and received at least another dispensing of rGH during the first year of treatment and were, therefore, included in the switching analysis.

The switching pattern of different rGH whithin 1 year after the ID showed that switch was not frequent (6.9%) (Figure S2 in Supplementary Material).

Considering all naïve rGH users, the median time of observation during the study period was 3.0 years.

**Figure 5:**
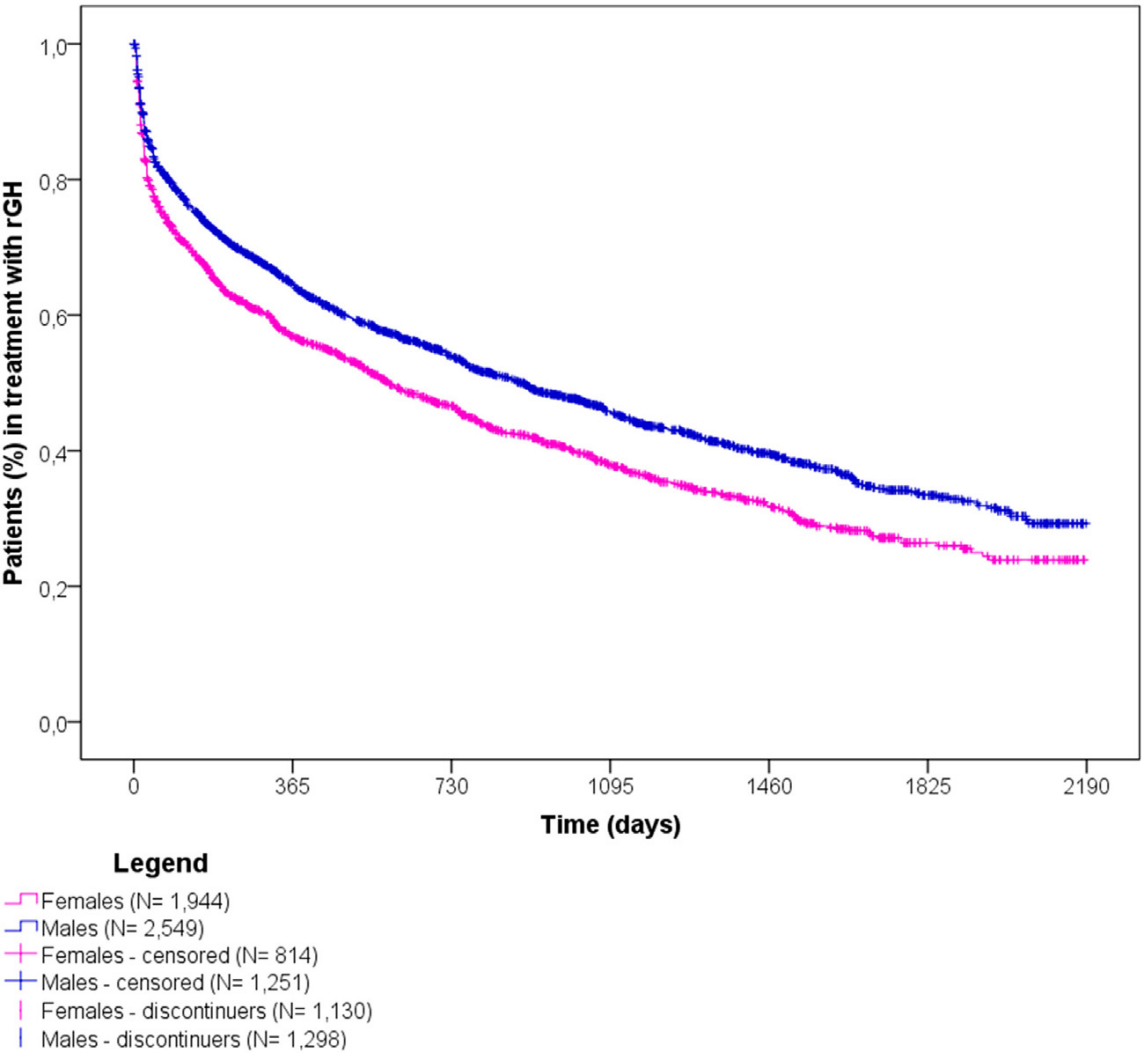
Time to discontinuation of recombinant growth hormone (rGH) therapy among naïve rGH users, stratified by sex.

More than half of rGH naïve users (*N* = 2,428; 54.0%) discontinued the therapy during observation time, more frequently in females than males (58.1 vs. 50.9%) (Figure 5). The overall median time to discontinuation was 25 months, but females discontinued earlier than males (median time to discontinuation = 19.5 vs. 29.7 months).

Among discontinuers, intermittent users were 39.3%, which means they restarted rGH therapy after at least 60 days of interruption. On average, they restarted rGH treatment 5 months after the discontinuation date. Of the remaining 60.7% discontinuers, 3.7 and 11.7% had at least one diagnosis of cancer and diabetes mellitus, respectively, while 9.7% were hospitalized mainly due to respiratory failure, and cardio- or cerebrovascular events. Finally, 7.9% were 15–17 years old at the time of discontinuation (data not shown).

**Figure 6:**
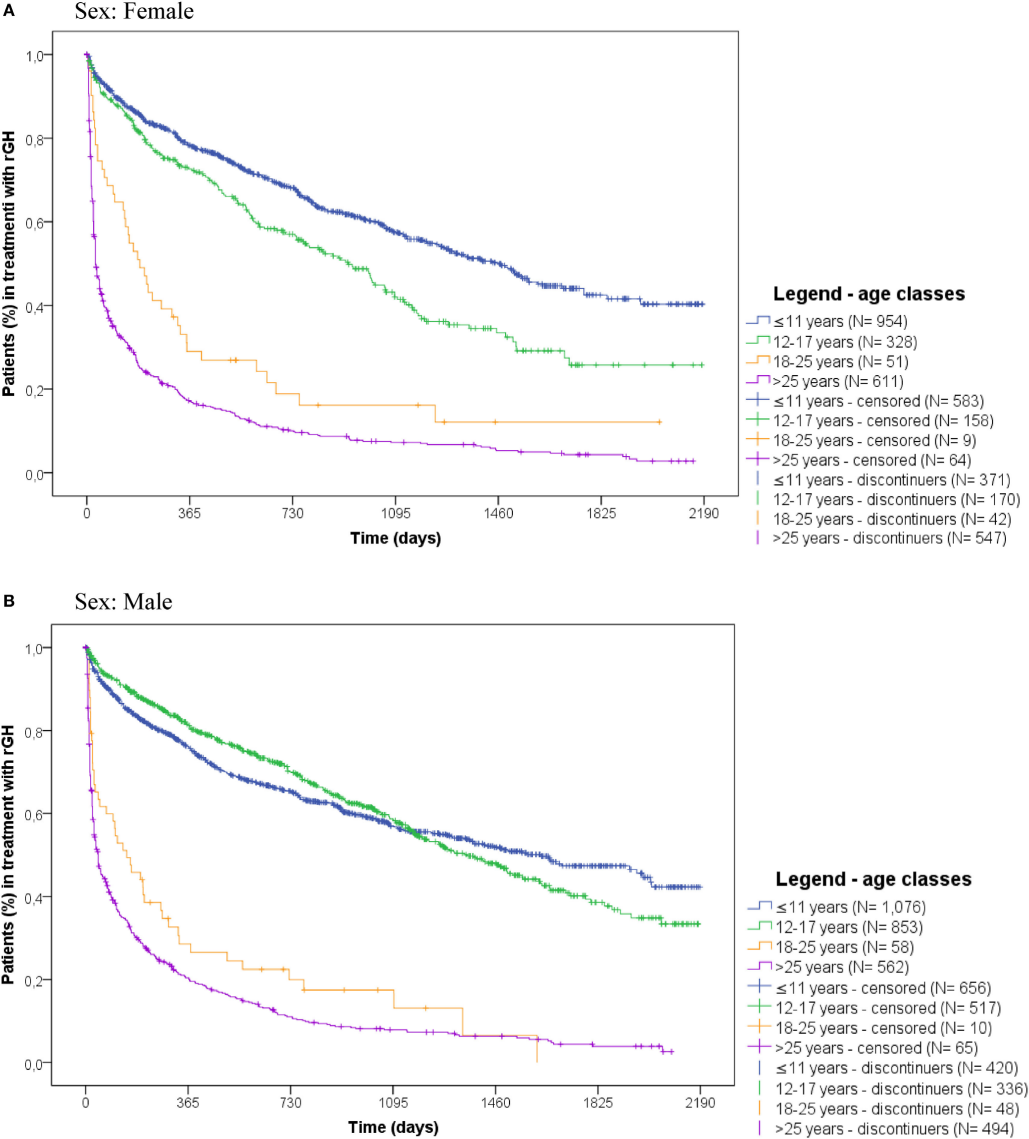
Time to discontinuation of recombinant growth hormone (rGH) therapy among naïve rGH users, stratified by sex and age classes. **(A)** Sex: female, **(B)** sex: male.

Stratifying by sex and age classes, in both sexes, naïve users >25 years old discontinued more frequently (males: *N* = 494 on 562, 87.9%; females: *N* = 547 on 611, 89.5%) and earlier than other age classes (median time to discontinuation: males = 1.5 months; females = 1 month). Naïve female users between 12 and 17 years old discontinued more frequently and earlier (*N* = 170 on total 328; 51.8%, median time to discontinuation = 30.9 months) than males of the same age class (*N* = 336 on total 853; 48.4%, median time to discontinuation = 45.3 months) (Figure 6).

**Figure 7:**
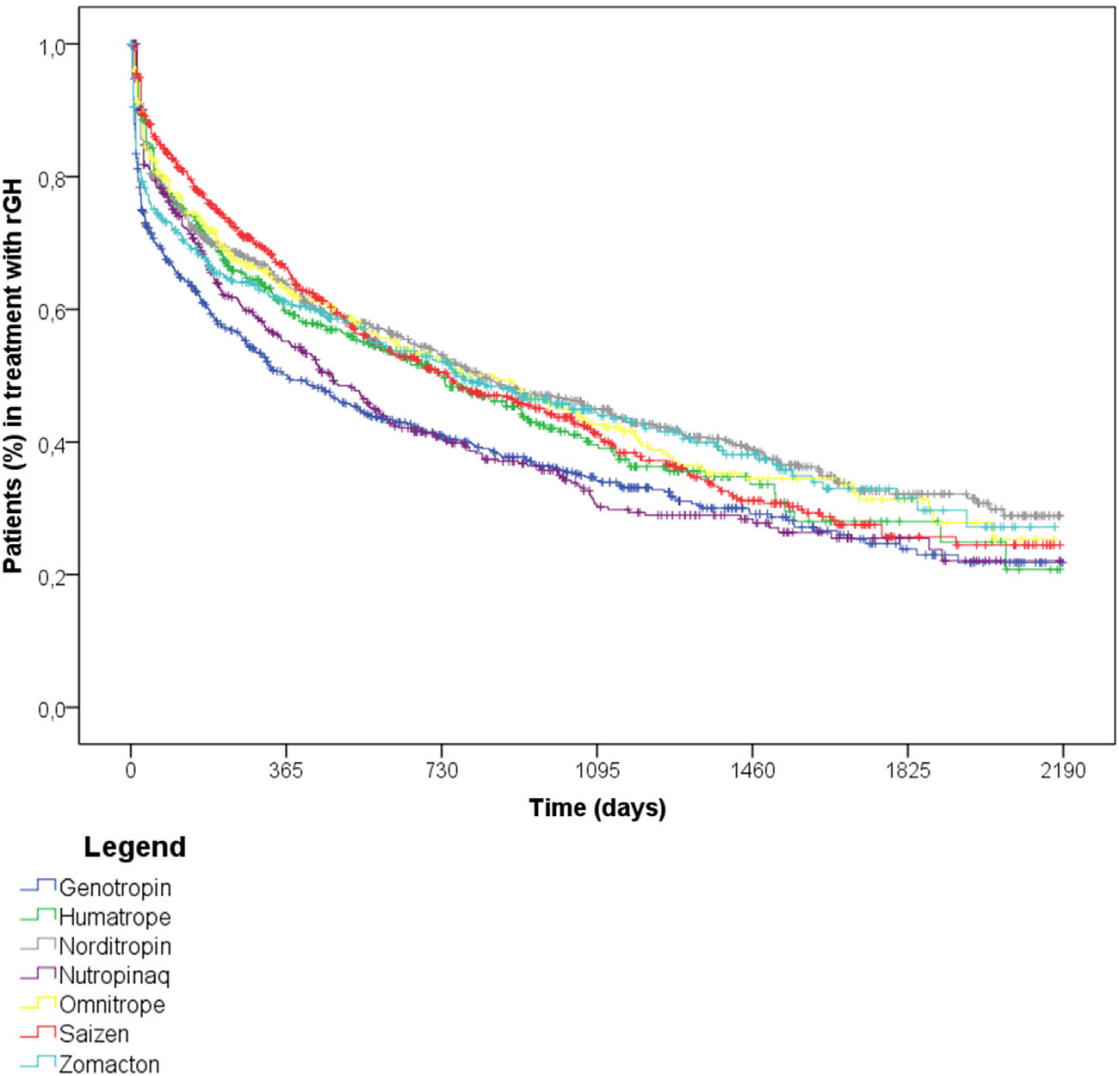
Time to discontinuation of recombinant growth hormone (rGH) therapy among naïve rGH users, stratified by medicinal product.

The log-rank test highlighted a statistically significant difference (*p*-value < 0.05) in the treatment persistence across various medicinal products (Figure 7), but no statically significant differences (*p*-value > 0.05) was observed in the treatment persistence of biosimilar rGH (Omnitrope^®^) vs. other medicinal products (i.e., Humatrope^®^, Norditropin^®^, Omnitrope^®^, Saizen^®^, Zomacton^®^).

Considering only the first year after ID, results from the sensitivity analysis showed that discontinuation was frequent (*N* = 1,501; 39.9%), especially among naïve users >25 years (*N* = 831 on total 1,013; 82.0%), thus confirming results from the main analysis, and much lower in those <18 years (22.6%) (data not shown).

The analysis on treatment persistence using a maximum allowed gap of 2 years showed that 22% (*N* = 989) of naïve users were discontinuers (data not shown).

**Figure 8:**
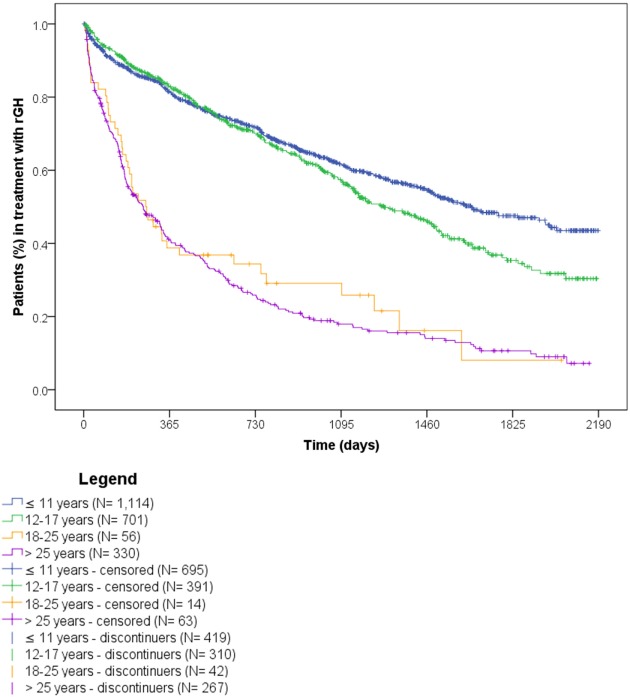
Time to discontinuation of recombinant growth hormone (rGH) therapy among naïve rGH users affected by growth hormone deficiency, stratified by age classes.

Considering only naïve rGH users affected by GH deficiency, 47.2% of them discontinued the treatment during follow-up (*N* = 1,038). Discontinuation was more frequent among naïve users >25 years old (*N* = 267 on total 330; 80.9%) and between 18 and 25 years old (*N* = 42 on total 56; 75.0%), than in those younger than 18 years (*N* = 729 on total 1,815; 40.2%) (Figure 8).

## Discussion

This population-based study is the first one exploring the pattern of rGH use in outpatient setting in a wide cohort of patients from six Italian Regions over six observation years. Our results highlight that the prevalence of rGH users slightly increased in the last years, up to 0.3 per 1,000 inhabitants, in line with the results from the National report of rGH users (6), which documented also a substantial heterogeneity across different Italian Regions. The topic has been previously investigated by Spandonaro et al. (4). Instead of the typical Italian “north–south” gradient observed for the health-care service utilization, results from this study showed that no pattern of rGH consumption could be made out across the Country. The authors suggested that variability in rGH consumption may be due to difference in prescribing appropriateness and to drug waste due to the different available devices for rGH administration. Although the variability observed by Spandonaro et al. may be justified by a different age composition of the population, our results from the age-adjusted prevalence of use still highlight a remarkable geographic heterogeneity. The migration of patients toward areas with a higher density of excellence centers for both the diagnosis and the treatment of growth disturbances may be a reason influencing the geographical variability in rGH consumption. However, the prescribing centers are well distributed across the whole Country and the patients are more likely to choose the closest endocrinology center, due to the frequency of the scheduled follow-up visits and to the storage conditions required by rGH (i.e., low temperature). Furthermore, the dispensing of rGH is carried out by the pharmacies of the patients’ city, thus making it more difficult for a patient to receive rGH outside the Region of residence.

Spandonaro et al. (4) also suggested that such a relevant geographical variability in rGH use may reflect the potential waste of drug, due to different combinations of dosing regimens and available devices. In detail, rGH is available on the market in the form of several devices, such as conventional syringes with needle, manual or automatic electronic devices self-injection pens, needle-free devices, with different characteristics in terms of ease of use, lack of pain during injection, electronic tracking system to monitoring treatment adherence, minimum dosage that can be administered, requested storage temperature, etc. The above mentioned study (4) suggested that these characteristics may influence the adherence to therapy, especially in younger patients, as well as the quantity of device-related wasted drug and the possibility of using all the rGH before the expiration date (8, 9, 22–24).

As documented by the national report on medicine consumption in Italy (25), the uptake of biosimilar rGH on the market was rather low (7.8%), which is much lower than that previously reported for biosimilars of epoetin alpha and granulocyte colony-stimulating factors (16, 17). Although biosimilar purchase cost is at least 20–30% lower than the reference product, the difference between biosimilar and reference products rGH prices is much higher than that of other biological drugs. The price difference may, therefore, not be a reason for such a low biosimilar rGH uptake. Furthermore, opposite trends in the use of biosimilar rGH were observed across centers, probably due to different center-specific health policy interventions adopted to promote biosimilars use, as already previously described (17). Based on the specific loco-Regional adopted approach, biosimilar should be prescribed to naïve users (whenever they represent the cheapest option) or clinicians are charged with the cost of the prescribed drug, in case it is not the cheapest and no justifications are provided or a yearly minimum thresholds of biosimilar use is established or the use of the cheapest drug is simply recommended. The geographical variability in biosimilar rGH uptake may be influenced by specific tender procedures put in place by each hospital or LHU or Region, which also vary over time. Other potential reasons for the heterogeneity in biosimilar rGH use are loco-regional differences in patients’ access to different rGH, different tender procedures for originators and biosimilars purchase by public structures, and the still ongoing skepticism about biosimilars (2), especially when target patients are pediatric as for rGH.

We were able to identify the indication for rGH use in 54% of naïve users. Most of them (88.8%) were affected by GH deficiency, as confirmed by previous data from the National report of rGH users (6).

Despite the position paper from the Italian Medicines Agency (26) and the specific loco-regional policy interventions, recommending that prescribers should prefer biosimilars in naïve patients, results from this study highlight that most of the naïve rGH users received a reference product at the ID.

This study shows that 0.8% of naïve rGH users had at least one diagnosis of benign neoplasm (in most cases, referred to pituitary gland, which lead to GH deficiency) within 6 months prior to the ID, while 1.3% of naïve rGH users had at least one registered diagnosis of malignant neoplasm. The Summaries of Product Characteristics of all drugs containing rGH contraindicate the use of rGH *when there is any evidence of activity of a tumor*, probably due to the mitogenic activity of rGH (27), which could, therefore, increase the risk of developing a recurrence of tumor (28–30). It is, therefore, likely that the registered malignant neoplasms referred to previous conditions or to neoplasms in remission.

In Italy, rGH prescription is based on clinical, biochemical, radiological, and genetic parameters and is reimbursed by NHS in adults in case of severe GH deficiency, and for pediatric patients during their evolutive age (i.e., until the final height is reached) for GH deficiency, in children born SGA, for short stature due to Turner or Prader–Willi syndrome, CKD, and SHOX deficiency.

Given the physical and psychological advantages of rGH therapy during the evolutive and transition age (i.e., the period between linear growth completion and 25 years old) (7, 31–33), no interruption should occur as rGH treatment aims at achieving full adult reproductive and somatic development, which occurs later in men than women and depends on GH levels (34, 35).

In adults, there are no specific clinical signs offering the same diagnostic certainty as the growth impairment occurring in children. Patients with adult-onset GH deficiency may exhibit increased fat mass, osteopenia, dyslipidemia, insulin resistance, altered cardiac structure, and function and consequential reduced quality of life (36). The final goal of rGH therapy in adults is to improve quality of life and reduce the cardiovascular risk (31).

Both in transition age and adulthood, the use of rGH is strictly dependent on the severity of GH deficiency and on the results of specific laboratory tests.

Discontinuation of rGH therapy occurred in 54.0% of naïve users. Results showed that medicinal products did not influence the discontinuation of rGH therapy, as no statistically significant differences were observed for biosimilar vs. originator rGH. In general, females discontinued earlier than males, probably due to their earlier hormonal and physical development, as confirmed by the higher percentage of discontinuers among females between 12 and 17 years old vs. males of the same age class. Naïve users >25 years old discontinued more frequently than other age classes, and these data were confirmed in the subgroup analyses on naïve users affected by GH deficiency. In general, discontinuation of rGH therapy may be due to different reasons. Our results showed that most of discontinuers were intermittent users (39.3%), thus highlighting temporary interruptions of rGH use, probably due to reduced patients’ compliance or to a lack of perceived benefit from rGH therapy. Another reason for discontinuing rGH therapy may be the occurrence of adverse reactions. More than 25% of discontinuers had a new diagnosis of diabetes or cancer or has been hospitalized at least once after discontinuation, suggesting the onset of specific conditions may be related to the low persistence to treatment. Specifically, cancer is enlisted among contraindications of rGH therapy, while diabetes, respiratory failure, and cardio- and cerebrovascular events are described in the Summary of Product Characteristics as undesirable effects of rGH use. Finally, 7.9% discontinued at the age of 15–17 years, probably due to final height achievement, not requiring further rGH use. The rest of discontinuers (27.7%) were likely to be related to lack or loss of rGH efficacy or to lack of patients’ compliance, as confirmed by the 17.9% of early discontinuers (i.e., discontinued within 6 months after the ID), most of whom were adults, aging ≥50 years (data not shown). It has to be also considered that: (i) the use of rGH during transition age is strictly dependent on the severity of GH deficiency and on the results of specific laboratory tests; (ii) the parameters based on which patients were eligible to rGH treatment changed over time, thus patients previously receving rGH may not be eligible thereafter. Further evaluations on the reasons for the discontinuation of rGH were not possible due to the lack of specific information in the available administrative databases. The time to discontinuation analyses were conducted using DDD, which refers to adults. Since doses are around fourfold or fivefold higher in pediatric patients than in adults, the analyses may underestimate treatment discontinuation in pediatric patients.

### Strengths and Limitations

One of the main strengths of this study is the availability of data about the rGH dispensing in six large Italian geographic areas over a 6-year observation period. Considering that biosimilar rGH was first marketed in 2006, we were able to investigate the long-term impact of its marketing on the pattern of rGH use.

Some rGH dispensings may not be captured in administrative databases (i.e., dispensing to inpatients), but study results are unlikely to be influenced by this limitation.

Part of naive users identified might be not primary naive users (i.e., first-ever users of the drug), but patients with previous exposure to rGH who underwent long-term interruptions (>1 year) of the rGH therapy, in line with the results from the persistence analysis. Using data from the administrative databases as a proxy, the identification of the indication of rGH use was possible for around 54% of naïve rGH users.

Further evaluations on the reasons for the observed geographical variability in rGH use were not possible due to the lack of such information in the available administrative databases, while potential reasons for the treatment discontinuation were investigated using dispensing databases, diagnosis at hospital discharge, and reasons for health-care service payment exemptions.

Even though our results may not be generalized to the whole national setting, data confirm those from the Italian national report on medicine consumption and with the report from the “National register of rGH users” corresponding to the study period.

## Conclusions

In recent years, a remarkable geographical variability in the prevalence of rGH use was observed. The proportion of biosimilar rGH users slightly increased over time but was rather low, as compared to other biosimilars (e.g., epoetin alpha and filgrastim), and likewise heterogeneous across different geographic areas, probably due to the different health-care policies adopted at a loco-regional level. Furthermore, the discontinuation or rGH therapy was frequent, but no statistically significant differences were observed for biosimilar vs. originator rGH.

Since rGH ranks first for the pharmaceutical expenditure concerning hormonal preparations in Italy, administrative health-care databases may represent a valid tool to continuously and rapidly monitor how this drug is used in routine care and may support decision makers in the adoption of effective post-marketing monitoring to guarantee greater economic saving than only promoting the lowest cost rGH.

## Ethics Statement

All procedures performed in this study were in accordance with the Ethical Standards of the Ethical Committee of the Academic Hospital of Messina (the study was approved on the 21st July 2014, minutes n.9/2014), according to the current national law, and with the 1964 Helsinki declaration and its later amendments or comparable ethical standards. The manuscript does not contain clinical studies, and all patient data were fully anonymized. For this type of study, formal consent is not required.

## Author Contributions

Study concept and design: GIAT. Acquisition of data (Caserta LHU): DT. Acquisition of data (Palermo LHU): MP and SS. Acquisition of data (Tuscany): RG. Acquisition of data (Treviso LHU): AC. Acquisition of data (Umbria LHU): GIUT. Acquisition of data (Lazio): VB and FT. Data management: FG and VI. Analysis and interpretation of data: IM, YI, GIAT, SC and PC. Preparation of manuscript: IM, YI, GIAT, GIUT, AA, and FT.

## Conflict of Interest Statement

GIAT declares his participation to advisory boards on biosimilars, organized by Sandoz and Hospira; furthermore, he coordinates a pharmacoepidemiology research team at the University of Messina, which receives research grants for projects that are not related to the topic of the paper. IM, YI, FG, VI, AC, DT, RG, SC, MP, SS, PC, GIUT, FT, VB, and AA declare that they have no conflicts of interest.
