# Supplementary material for: Pattern of Use of Biosimilar and Originator Somatropin in Italy: A Population-Based Multiple Databases Study During the Years 2009–2014
Source: Front Endocrinol (Lausanne). 2018 Mar 13;9:95. doi: 10.3389/fendo.2018.00095 (PMC5859012; doi:10.3389/fendo.2018.00095)
Supplement: Supplementary file 1 [file table_1.PDF]

Supplementary Material Table 1 Available rGH (ATC: H01AC01) in Caserta, Lazio, Palermo, Tuscany, Treviso and Umbria databases

Brand name (MA date)	Type of rGH	Indication for use							Dosage	Price[a] (in €)	Notes
		Children						Adults			
		**insufficient secretion of GH** (0,025–0,035 mg/kg/daily)	**growth disturbance due to Turner syndrome** (0,045–0,050 mg/kg/daily)	**growth disturbance due to CKD** (0,045–0,050 mg/kg/daily)	**Prader-Willi syndrome** (0,035 mg/kg/daily)	**growth disturbance in short children born SGA** (0,035 mg/kg/daily)	**growth failure associated with SHOX deficiency** (0,045-0,050 mg/kg/daily)	**replacement therapy for severe GH deficiency** (0,15–0,3 mg/daily)			
Genotropin (1998)	Reference product	✔	✔	✔	✔	✔		✔	Goquick 1 pen 12 mg	473,23	• Before reconstitution: store at 2-8°C, or at <= 25°C (for maximum 6 months);<br>• Automaticly ready-mixed solution (no handling);<br>• Goquick device is single-use (no store in refrigerator) and allows minimum dose increments of 0,05 mg
									Goquick 1 pen 5,3 mg	232,24	
									Miniquick 7 cartridges 0,2 mg	52,86	
									Miniquick 7 cartridges 0,4 mg	114,80	
									Miniquick 7 cartridges 0,6 mg	151,76	
									Miniquick 7 cartridges 0,8 mg	214,79	
									Miniquick 7 cartridges 1 mg	279,51	
									Miniquick 4 cartridges 1,2 mg	180,28	
									Miniquick 4 cartridges 1,4 mg	221,69	
									Miniquick 4 cartridges 1,6 mg	257,89	
									Miniquick 4 cartridges 1,8 mg	295,08	
									Miniquick 4 cartridges 2 mg	332,36	
Humatrope (1997)		✔	✔	✔		✔	✔	✔	6 mg solution for injection	260,37	• Reduced waste of drug
									12 mg solution for injection	372,67	

									24 mg solution for injection	745,34	
Norditropin (1999)		✓	✓	✓		✓		✓	SimpleXx 5 mg/1,5 ml	192,84	• After reconstitution: possibility to be stored at <= 25 °C for maximum 3 weeks;<br>• Reduced waste of drug
									SimpleXx 10 mg/1,5 ml	685,76	
									SimpleXx 15 mg/1,5 ml	578,51	
									Nordiflex 1 pen 5 mg	192,84	
									Nordiflex 1 pen 15 mg	578,40	
Saizen (2001)		✓	✓	✓		✓		✓	8 mg 1 cartridge click-easy	345,33	• Dose-memory function (adherence monitoring);<br>• Hidden needle and different auto-injectors available;<br>• Before reconstitution: possibility to be stored at <25°C;<br>• After reconstitution: possibility to be stored at <= 25°C for maximum 7 days;<br>• Reduced waste of drug
									6 mg 1 cartridge	247,48	
									1,33 mg soluction for injection	54,86	
									12 mg/1,5 ml cartridge solution for injection	494,96	
Nutropinaq (2001)		✓	✓	✓				✓	10 mg 2 ml 1 cartridge	327,05	• Dose-memory function;<br>• Needle shield;<br>• Reduced waste of drug
Zomacton (1994)		✓	✓						4 mg solution for injection	171,29	• No needle;<br>• Reduced waste of drug
Omnitrope (2006)	Biosimilar	✓	✓	✓	✓	✓		✓	5 mg/1,5 ml cartridge	126,62	
									10 mg/1,5 ml cartridge	240,55	
									Surepal 1 cartridge 5 mg/1,5 ml	126,62	
									Surepal 1 cartridge 10 mg/1,5 ml	240,55	

									Surepal 1 cartridge 15 mg/1,5 ;ml	360,83	

Legend: rGH= recombinant growth hormone (somatropin); MA= market authorization; GH= growth hormone; CKD= chronic kidney disease; SGA= small for gestational age.

[a]Data source: Farmadati: http://www.farmadati.it/ - accessed: December 3rd, 2016.
Zimoser® was available in Italy between June 2010 and October 2013, but no dispensings were found in the participating databases.
Auxonorm® was available in Italy until August 2000.
Somatropin Biopartners® was not marketed in Italy, during the study period.
