# Supplementary material for: Pattern of Use of Biosimilar and Originator Somatropin in Italy: A Population-Based Multiple Databases Study During the Years 2009–2014
Source: Front Endocrinol (Lausanne). 2018 Mar 13;9:95. doi: 10.3389/fendo.2018.00095 (PMC5859012; doi:10.3389/fendo.2018.00095)
Supplement: Supplementary file 2 [file table_2.PDF]

Supplementary Material Table 2 Healthcare policy interventions regarding biosimilar rGH use in the participating centres

Centre	Date	Health policy interventions	Description of health policy interventions
**Tuscany**	**28.07.2014**	Regional Decree n. 644 – Identification and assignment of Local Health Units' goals regarding the appropriateness of prescriptions for the year 2014 [Regione Toscana. Deliberazione della Giunta Regionale 28 luglio 2014, n. 644. Regione Toscana - BURT del 06/08/2014. 2014. http://www.regione.toscana.it/documents/10180/11864888/PARTE+II+n.+31+del+06.08.2014.pdf/ae79493b-3af0-451b-986d-c21ae3d7c3ec]	Unit doses of biosimilars containing rGH (ATC H01AC01), distributed by direct/territorial dispensing and hospital consumption, must be greater than or equal to 50%, in relation to total distributed unit doses of biological products containing somatropin (ATC H01AC01).
**Veneto (Treviso)**	**19.12.2012**	Note n. 576546. Recommendations from the Regional Commitee for rGH monitoring about prescription of originators and biosimilar rGH [Regione Veneto. Indirizzi prescrittivi per l'impiego delle specialità e biosimilari contenenti GH espressi dalla Commissione Regionale GH. Nota prot. n. 576546 del 19.12.2012. 2012. https://www.regione.veneto.it/c/document_library/get_file?uuid=a19f5eae-c261-4fee-8dd3-0ebb6d2519ef&groupId=10793]	In light of a substancial homogeneity concerning indications for use, the use of drugs containing rGH at the lowest cost is recommended.<br><br>Given the comparability, in terms of efficacy and safety, of the drugs containing rGH, clinicians should prefer the cheapest drugs, after taking into account indications for use, patient's compliance, therapeutic continuity, tolerability of excipients. Considering adult patients, the devices providing the less waste of product should be preferred.
	**20.12.2013**	Regional Decree n. 2533 – Identification of goals to be reached by Local Health Units and public hospitals for the year 2014 [Regione Veneto. Deliberazione della Giunta Regionale n. 2533: Determinazione degli obiettivi di salute e di funzionamento dei servizi per le Aziende ULSS del Veneto, l'Azienda Ospedaliera di Padova, l'Azienda Ospedaliera Universitaria Integrata di Verona e per l'IRCCS ''Istituto Oncologico	The total Defined Daily Dose (DDD) biosimilars rGH, as hospital consumptions or distributed by direct/territorial dispensing, on the total DDD of drugs belonging to the ATC H01AC01 must be greater than or equal to 14%.

		Veneto''—anno 2014. 20/12/2013. 2014. http://bur.regione.veneto.it/BurvServices/pubblica/DettaglioDgr.aspx?id=265138]	
	**11.03.2014**	Regional Decree n. 248 – Identification of specific procedures for prescription, dispensing and monitoring of rGH [Regione Veneto. Deliberazione della Giunta Regionale n. 248: Attivazione dell'applicativo regionale informatizzato per la prescrizione, la dispensazione e il monitoraggio dell'ormone della crescita (GH). 2014. http://bur.regione.veneto.it/BurvServices/Pubblica/DettaglioDgr.aspx?id=270622]	A regional electronic form for rGH prescription is created, allowing the collection of specific data on treated patients, the reimbursement by the NHS based on "Distribuzione Per Conto". The final goal is to correctly monitor rGH prescriptions and costs.
**Sicily (Palermo)**	**08.01.2014**	Administrative Decree for the inclusion of drugs in "distribuzione per conto [Regione Sicilia. D.A. Approvazione dell'Accordo per la Distribuzione per conto (DPC) dei farmaci inclusi nel prontuario terapeutico ospedaliero (PHT) del 8/01/2014. 2014. http://sanita.e-shark.it/downloads/aggiornamenti/DPC_D.A.8.1.14.pdf]	Drug classes identified by ATC codes B03XA, L03AA and H01AC01 are included in "distribuzione per conto" (DPC); for these therapeutic classes, the appropriateness form is available.
	**28.04.2014**	Administrative Decree n. 540/14. Recommendations regarding the use of originators and biosimilar at the lowest cost – further explanations [Regione Sicilia. D.A. n. 540/14 Misure volte a promuovere l'utilizzo dei farmaci Originatori o Biosimilari a minor costo di terapia. Circolare esplicativa del 28/04/2014. 2014. http://pti.regione.sicilia.it/portal/page/portal/PIR_PORTALE/PIR_LaStrutturaRegionale/PIR_AssessoratoSalute]	For drug classes identified by ATC codes B03XA, H01AC01 e L03AA: "Prescribers may fill the therapeutic plan attached to Administrative Decree n. 540/14, describing the reason why a drug with higher cost, other than a biosimilar, is chosen"; the therapeutic plan should be attached to the prescription of the National Health Service. In case the clinician prescribes the drug with higher cost, the pharmacist will verify if the therapeutic plan is addressed to a naïve patient.
**Lazio**	**29.08.2014**	Regional Decree n. G12190. rGH treatment: recommendations [Regione Lazio. Trattamento con ormone somatotropo (GH): linee di indirizzo ed	Dispensings of rGH are included in "distribuzione per conto" (DPC).<br><br>Considering a careful evaluation of efficacy and safety of drugs containing rGH and the sustainability of the National

		aggiornamento dell'elenco delle strutture autorizzate. Determinazione n. G12190 del 29.08.2014. 2014. http://pubbur.ised.it/PublicBur/burlazio/FrontEnd]	Health System, the use of the cheapest drug is recommended, except for relevant clinical reasons (therapeutic continuity, tolerability of excipients). These reasons have to be documented in the therapeutic plan.
	**12.05.2015**	Decree n. G05686 from the Health and Social Committee of Lazio Region [Regione Lazio. Costituzione Gruppo di Lavoro "Farmaci Biosimilari". Determinazione della Direzione Regionale Salute e Integrazione Sociosanitaria del Lazio n. G05686 del 12.05.2015 e s.m.i. 2015. http://www.regione.lazio.it/rl_main/]	A specific "Working Group on Biosimilars" is created.

Legend: rGH= recombinant growth hormone (somatropin); ATC= Anatomical Therapeutic Chemical classification system.
