# Supplementary material for: Pattern of Use of Biosimilar and Originator Somatropin in Italy: A Population-Based Multiple Databases Study During the Years 2009–2014
Source: Front Endocrinol (Lausanne). 2018 Mar 13;9:95. doi: 10.3389/fendo.2018.00095 (PMC5859012; doi:10.3389/fendo.2018.00095)

Supplementary Material Image 1 Distribution of different rGH in naïve users in the six participating centres

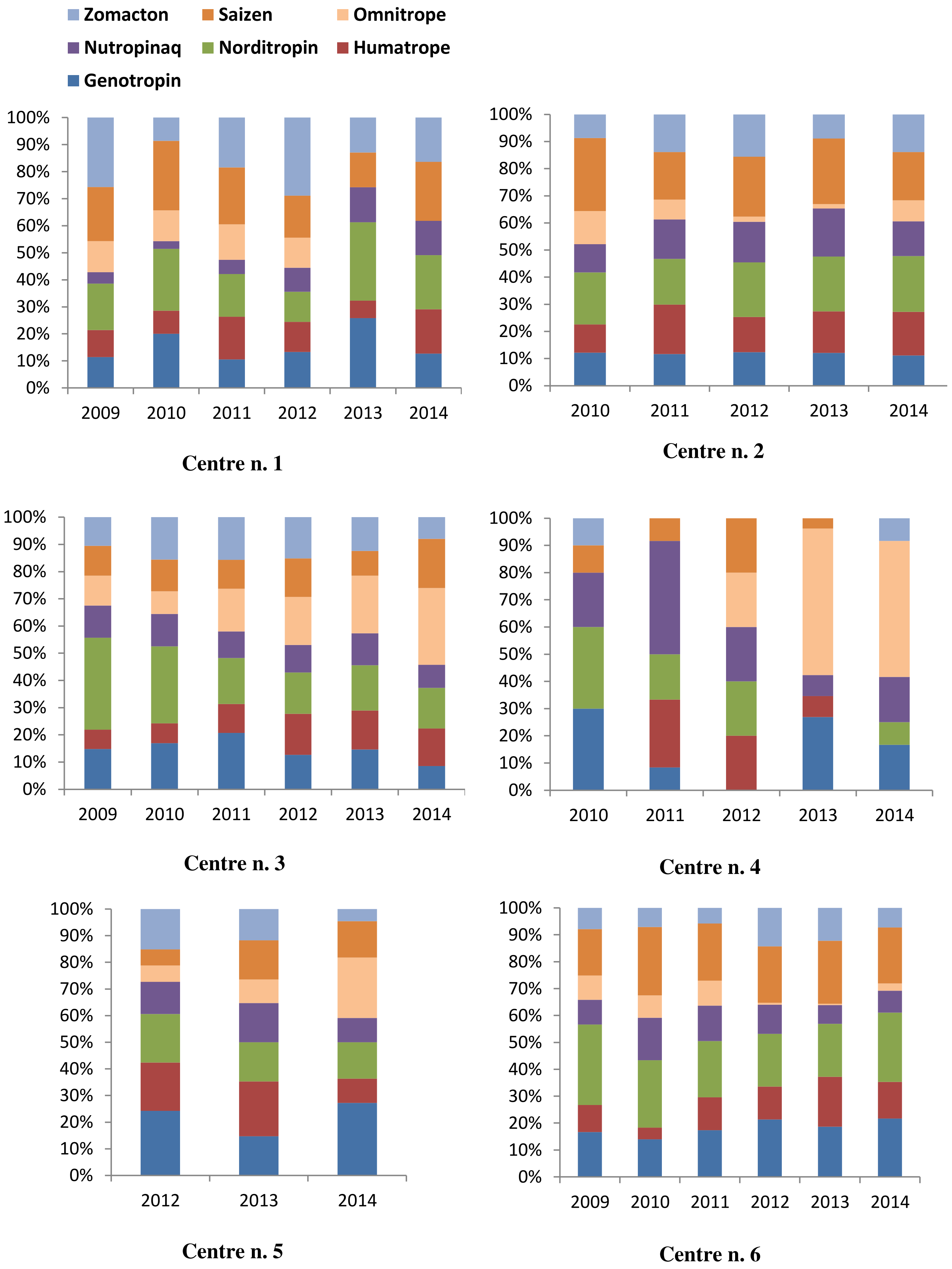

Supplement: Supplementary file 3 [file image_1.PDF]
