# Supplementary material for: Pattern of Use of Biosimilar and Originator Somatropin in Italy: A Population-Based Multiple Databases Study During the Years 2009–2014
Source: Front Endocrinol (Lausanne). 2018 Mar 13;9:95. doi: 10.3389/fendo.2018.00095 (PMC5859012; doi:10.3389/fendo.2018.00095)
Supplement: Supplementary file 4 [file image_2.PDF]

Supplementary Material Image 2 Switching pattern of different rGH during the first year of treatment in all centres

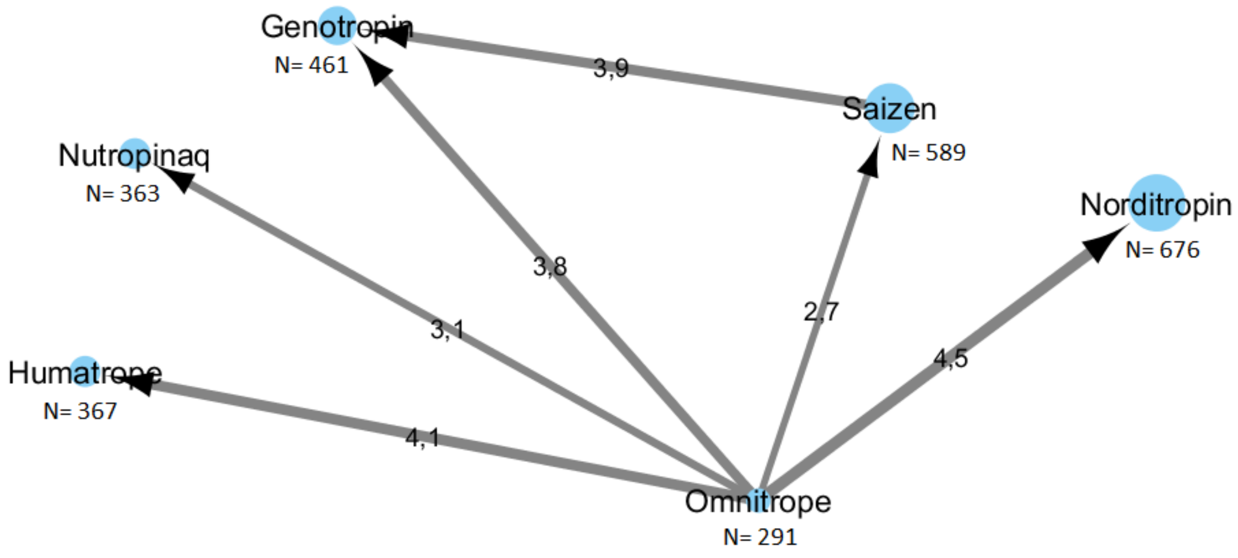

The size of the nodes indicates the number of users; size of the arrows indicates the proportion of users that switch from one product to the other; only the first switch after the ID was considered. Percentages were calculated based on the total users of the starting rGH (minimum 2%).
